# Does a competitive voucher program for adolescents improve the quality of reproductive health care? A simulated patient study in Nicaragua

**DOI:** 10.1186/1471-2458-6-204

**Published:** 2006-08-07

**Authors:** Liesbeth E Meuwissen, Anna C Gorter, Arnold DM Kester, J Andre Knottnerus

**Affiliations:** 1 Instituto CentroAmericano de Salud (ICAS), Postal 2234, Managua, Nicaragua; 2 The Netherlands Institute for Health Services Research (NIVEL). Postbus 1568, 3500 BN Utrecht, The Netherlands; 3 London School of Hygiene and Tropical Medicine, Keppel Street, London WC1E 7HT, UK; 4 Department of Methodology and Statistics, University of Maastricht, Postbox 616, 6200 MD Maastricht, The Netherlands; 5 Department of General Practice, University of Maastricht, PB 616, 6200 MD Maastricht, The Netherlands; 6 Health Council of the Netherlands, Postbus 16052, 2500 BB Den Haag, The Netherlands

## Abstract

**Background:**

Little is known about how sexual and reproductive (SRH) health can be made accessible and appropriate to adolescents. This study evaluates the impact and sustainability of a competitive voucher program on the quality of SRH care for poor and underserved female adolescents and the usefulness of the simulated patient (SP) method for such evaluation.

**Methods:**

28,711 vouchers were distributed to adolescents in disadvantaged areas of Managua that gave free-of-charge access to SRH care in 4 public, 10 non-governmental and 5 private clinics. Providers received training and guidelines, treatment protocols, and financial incentives for each adolescent attended. All clinics were visited by female adolescent SPs requesting contraception. SPs were sent one week before, during (with voucher) and one month after the intervention. After each consultation they were interviewed with a standardized questionnaire. Twenty-one criteria were scored and grouped into four categories. Clinics' scores were compared using non-parametric statistical methods (paired design: before-during and before-after). Also the influence of doctors' characteristics was tested using non-parametric statistical methods.

**Results:**

Some aspects of service quality improved during the voucher program. Before the program started 8 of the 16 SPs returned 'empty handed', although all were eligible contraceptive users. During the program 16/17 left with a contraceptive method (p = 0.01). Furthermore, more SPs were involved in the contraceptive method choice (13/17 vs.5/16, p = 0.02). Shared decision-making on contraceptive method as well as condom promotion had significantly increased after the program ended.

Female doctors had best scores before- during and after the intervention. The improvements were more pronounced among male doctors and doctors older than 40, though these improvements did not sustain after the program ended.

**Conclusion:**

This study illustrates provider-related obstacles adolescents often face when requesting contraception. The care provided during the voucher program improved for some important outcomes. The improvements were more pronounced among providers with the weakest initial performance. Shared decision-making and condom promotion were improvements that sustained after the program ended. The SP method is suitable and relatively easy to apply in monitoring clinics' performance, yielding important and relevant information. Objective assessment of change through the SP method is much more complex and expensive.

## Background

Nicaragua has one of the highest adolescent fertility rates of Latin America, with 119 births annually per 1000 young women aged 15–19. By the age of 19, 45% of the girls are either pregnant or have already given birth. High fertility rates are associated with low socio-economic status and low educational attainment [1]. In addition, adolescents experience high rates of unwanted pregnancy, illegal abortions; high maternal mortality rates, and are at high risk of contracting sexually transmitted infections (STIs), including HIV. These risks are closely connected with the low use of contraceptive methods among sexually active adolescents. Although 98% of the 15–19 year olds could name at least one contraceptive method, only 46% of the sexually active girls in this age group used such a method [1].

Major reasons for the low use of contraceptive methods among adolescents are lack of access to information and services on sexual and reproductive health (SRH) and a low quality of care. While obstacles to appropriate care have been documented extensively [2-5], there is an urgent need to better understand how to change the existing situation [6,7].

Evidence of interventions that have succeeded in improving the quality of SRH care for adolescents in existing health centres in Latin America is scarce. Indications existed that the competitive voucher program for sex-workers in Managua, Nicaragua, succeeded in improving the quality of care for these women [8,9], however this was never subject of specific research. ('Competitive' refers to the fact that a number of different providers are involved in the program, with consumers able to choose between them. This encourages the providers to compete to attract and retain voucher holders). Therefore, when the Central American Health Institute (ICAS) implemented a pilot voucher program between 2000 and 2002, designed to increase access to and quality of SRH care for poor and underserved adolescents, various aspects of the quality of care provided were closely monitored and evaluated. Though the program had impact on boys and girls, we focussed the evaluation on girls due to resource constraints and because we wanted to assess if girls could be supported to take control of their fertility, without requiring the consent of parents or partner.

The evaluation showed that voucher receipt increased use of SRH care among female adolescents (adjusted odds ratio 3.1, 95% confidence interval 2.5 – 3.9) and of contraceptives and condoms in specific groups [10]. Furthermore, girls were more satisfied with the quality of SRH care delivered through the voucher program, compared to care delivered without voucher [11]. Quality as perceived by the patients is an important aspect of quality, but not the only one the program aimed at influencing.

The objective of the simulated patient (SP) study reported here was to assess whether the technical quality, the content of the communication and the general treatment were improved by the voucher program, and whether any change was sustained after the intervention ended. The second aim of this exploratory study was to evaluate the usefulness of the SP method to evaluate such changes. SPs were sent to the participating clinics, before, during and after the intervention to request a contraceptive method. This paper reports on their experiences with the health care and our experiences with the SP method.

## Methods

### The intervention

The intervention took place in Managua, the capital of Nicaragua, one of the poorest countries of Latin America. Primary health services in Managua consist of public health centres run by the Ministry of Health, municipal public health centres, private clinics, and clinics run by non-governmental organisations (NGOs). Most participating clinics are staffed by two doctors, and in most larger clinics two doctors were allocated to receive adolescents.

Over fifteen months, 28,711 vouchers were distributed to adolescents aged between 12 and 20, at markets, outside schools and door-to-door in disadvantaged neighbourhoods. The only selection criterion was age. The vouchers gave free of charge access to SRH care in any of the four public, five private or ten NGO clinics contracted by ICAS, for one consult and a follow-up visit. The selection of clinics was based on suitability and proximity to the areas where vouchers were distributed. Identified clinics were invited to participate, and the price per consultation was negotiated based on their customary fees. The average price negotiated per consultation *and *follow-up visit was US$ 4.56. Adolescents were free to redeem their voucher in a clinic of their choice, and the clinics received reimbursement for each adolescent consultation with a voucher. The voucher program started with four clinics and new clinics were added periodically.

Vouchers were valid for three months and 20% of the vouchers distributed to girls were redeemed (boys 6%). This is a relatively high redemption rate considering the limited period over which the vouchers were valid and that a large percentage of the receivers was not yet sexually active. Among sexually active girls, 51% used their voucher, while among girls who were not yet sexually active, use was only 14% [10].

The voucher program addressed various aspects of quality of care. Doctors had to complete a standardized clinical form based on 'best practice' protocols, which guided them during each consultation. The form was designed to ensure that every adolescent was asked about their sexual activity; their need for information; their need for contraceptives; and was given a package with two condoms plus health education material on adolescence and STIs. Contraceptives, drugs for syndromic treatment of STIs and laboratory tests for pregnant girls were made available through the program. It was assumed that the competitive nature of the voucher program would prompt providers to improve the quality of services to attract more voucher users [8]. Doctors at participating clinics received an information manual and were obliged to attend an introductory meeting to learn about the program and its procedures. Furthermore, they were encouraged to attend a training course conducted over three mornings on 'adolescent friendliness' [12], counselling, adolescence and sexuality, contraceptives, and sexual abuse organized by the University of Nicaragua. Seventy percent of the doctors participated in at least one training session. In addition, clinic receptionists received training on 'adolescent friendliness'

### The design

In the SP method patients are 'standardized' making it possible to compare the performances of different doctors or of one doctor at different moments. The use of the SP method in developed countries has proven to be reliable, valid, feasible and acceptable [13-15]. The reported experiences in developing countries are promising [16-20]. To assess the individual performance of a doctor one has to use at least 8 to 10 SPs, but for the assessment of a group performance for one medical problem, one SP probably suffices [15,21]. To evaluate the intervention we measured the performance pre- during and post intervention. We selected the clinics as unit of analysis, because the intervention envisaged to improve the quality of care provided by the participating clinics.

All 19 participating clinics signed a contract of agreement that included information that the quality of care would be evaluated on a regular basis. They were not informed specifically on the methods to be used. The week before the intervention started in a certain clinic, a SP without a voucher was sent. During the course of the program, SPs were sent with a voucher to monitor the quality of care provided under the program. One to two months after the program ended, SPs without a voucher visited each clinic again.

The SPs were female adolescents active in NGOs involved in the voucher distribution, and university students participating in social work activities. They were contracted on a day-to-day basis and were paid a days' wage and enough money to pay for the consultation fees, if needed, and transportation costs. In total, 17 SPs took part in the survey. Their mean age was 18.7 years (range 16–22 years) and seven had secondary school education and 10 studied at the university. The mean number of doctors visited was 3.5 (range 1 – 12).

All SPs received a short training on the task to be performed. The objective of the study was explained and the girls were instructed to present a story that over the past three months they had formed a relationship with a boyfriend and were presently using withdrawal or periodic abstinence, but did not want to risk pregnancy. Furthermore, they had to ask for information on HIV/AIDS. All were told that they were free to accept or reject any information or services offered to them. If necessary, they were advised to decline vaginal examination by saying that they were currently menstruating.

The SPs were not accompanied to the clinic. If needed, the SP would make an appointment. After the consultation, the SPs were immediately interviewed by one of the two doctors of the research team at the ICAS office, using a standardized questionnaire. The answers were recorded by the interviewer on the questionnaire. The study was approved by the ethical review committee of ICAS.

### Measurement

The questionnaire contained 35 open-ended questions and included commonly used elements for evaluating quality of family planning services: choice of contraceptive methods, information given to users, technical competence, interpersonal relations, mechanisms to encourage continuity and appropriate organisation of services [22]. Questions included: Did the doctor explain a contraceptive-method? Which methods were explained to you? Which method was decided upon? Did the doctor orientate you about the method or did you feel he/she imposed the method? What did he/she tell you about the advantages and disadvantages of the method? Where you satisfied with the explanations given by the doctor? (The questionnaire is available on request).

The 21 criteria for evaluation are based on the program objectives, and were extensively discussed by the research team and defined before the analysis was performed. They are grouped into four categories. Two categories relate to contraception: A. method choice and B. continuity in use; category C. relates to prevention of STIs; and D. to the organisation of the services. The last category is composed of a selection of criteria that have been found to influence patients' perception of the quality of care, but do not relate directly to the doctors performance. A maximum score of 10 points could be attained if there was a positive response for all criteria in one category. The addition of the scores of the first three categories serves as an indicator of the quality of the consultation. For more details on the components of each category see Tables 1 and 2. The internal consistency for category A to C is high. Cronbachs alpha is respectively 0.67, 0.60 and 0.72. For category D the alpha is 0.26, which is not a surprise in view of the independency of the underlying criteria (e.g. waiting time, cleanliness and privacy are not necessarily associated, though all important).

### Data handling and analysis

Information on date of interview, clinic, and doctor visited was disconnected from the file, and records were put in a random order. Codification was done by one person, and checked by another. All data were entered twice in Epi-info 6.04 by two different data processors. Stata 7.0 (StataCorp, College Station, TX, USA) was used for further analysis.

The response to each criterion is tabulated in table 1 and 2. To guarantee equal representation of all clinics, mean scores per criterion were calculated for clinics visited more than once during a particular round. The Wilcoxon signed ranks test (paired design) was used to look for differences in performance before and during the intervention, and before and after the intervention. Because the sample size is small, associations with borderline significance (0.5 < p < 0.10) are also reported.

To get an indication as to whether doctors' characteristics influence their performance while counselling for contraception and STI/HIV, the sum scores of the three categories reflecting the quality of the consultation are calculated, at the three relevant time-points. Because the doctors are not necessarily the same at the different time-points, only comparisons per time-point are made. Per time-point, the non-parametric Mann Whitney test is used to assess the influence of doctors' gender and the Kruskal-Wallis test to assess the influence of the age group and the type of clinic on their responses. Since doctors had to complete a medical form for all patients with a voucher, this included the SPs. These forms were used to evaluate the SPs' performance and doctors' registration.

## Results

All clinics received SPs. In total 65 visits were made to 16 clinics before, 17 during and 15 after the intervention. Seventeen SPs visited 16 of the 19 participating clinics before the intervention. In one clinic, the SP was received by the counsellor instead of the doctor, one was sent too late and treated according to the voucher protocol, and one is missing for unknown reasons. During the intervention, 17 clinics were visited by 30 SPs with a voucher. One of the clinics was excluded due to administrative problems and one resigned from further participation. After the pilot, one clinic was temporarily closed and another permanently closed. Eighteen SPs visited the remaining 15 clinics.

Thirty-three of the 40 doctors who participated in the voucher program received at least one SP. Their characteristics are similar to the entire group of participating doctors. Six (*18%*) of the doctors worked in a public facility; 19 (*58%*) in an NGO clinic and 8 (*24%*) in a private clinic. Sixty-one percent are female and 39% younger than 35 years, broadly conforming to the gender and age profile of primary health care doctors in Nicaragua.

### The choice of the method (Table 1, part A)

Few SPs said they received counselling on all contraceptive methods. During the voucher program the number of methods presented was more frequently less than three than before the program (A2, 12/16 vs. 11/17 vs. p = 0.09). However, the number of barriers to use diminished. Barriers related to unnecessary diagnostic tests disappeared during the voucher program (A3, 12/16 vs. 17/17, p = 0.08), but returned after the program.

There was considerable variation in the methods recommended by the doctors. Some doctors promoted the condom for its safety or dual protection, where other doctors advised that condoms were unsafe, boyfriends would resist using them, or warned about possible allergic reactions. The same variation was seen in methods such as the intra-uterine device and monthly injectables. Before and after the program, some doctors recommended natural methods like periodic abstinence as best method for adolescents. The quality of the explanations given improved during the voucher program and remained better after the program ended (A5, p = 0.08).

Before the intervention started, most SPs indicated that it was the doctor who decided on the type of contraceptives to be used. Patients with a voucher reported more frequently that they made the choice jointly with the doctor (A4, 13/17 vs. 5/16, p = 0.02). The percentage of SPs reporting shared decision-making remained relatively high after the intervention (p = 0.03).

### Continuity in use (Table 1. part B)

Although all SPs were eligible contraceptive users, before the program eight of the 16 SPs left the consultation room without a contraceptive method or prescription. Six of these eight SPs were told to come back on the first day of their next menstrual cycle. One doctor preferred to wait for the results of unnecessary laboratory tests (e.g. liver function tests, urine sediments) and one advised periodic abstinence (the 'rhythm method').

During the voucher program, 94% of the girls left the clinic with a contraceptive method (B1, p = 0.01). This improved practice continued after the program ended (p = 0.10), but the frequency of follow-up appointments decreased (B3, p = 0.08).

### Information on STIs/HIV (Table 1, part C)

Although the percentage of doctors discussing STI/HIV-risks during a consultation increased, a considerable number of patients did not receive the desired information. More than 10% of the SPs in each round felt the doctor was in a hurry to end the consult. Some felt pressured to the extent that they did not ask their question on STIs.

Although in less than half of the consultations before, during and after the intervention SPs were satisfied with the explanations provided, patients seen after the intervention more frequently received correct information than before (C2, p = 0.10) and condom promotion increased from 53% to 93% (C5, p = 0.03).

There was considerable variation in the content of the information on prevention on STIs/HIV. Some doctors gave good and reliable information where others confined themselves to observations like: "One has to know the person with whom one is with"; "The only way to prevent AIDS is not to sleep with someone who has AIDS"; "One should not use clothes from other persons"; or "If someone's partner is sweating and having weight loss he might have AIDS".

### Organisation of the clinic (Table 2)

No clear differences in the organisational aspects of the clinic could be established, except that privacy during consultation appeared better respected after than before and during the intervention (p = 0.08).

### Handling of voucher users

Patients with a voucher were not always treated according to the contractual agreements. SPs reported that receptionists sometimes completed considerable parts of the medical forms and that other patients were favoured in the waiting room.

Experiences of the SPs with vouchers suggest that some private clinics tried to make as much money as possible out of the program by erroneous declarations, and by selling or withholding the materials provided by the program.

### Other factors influencing the results (Table 3)

The gender of the doctor was strongly associated with the results. Female doctors scored better than male doctors before, during and after the intervention. Male doctors improved by a greater degree during the voucher program. However, these changes were not sustained except for the category on STI/HIV prevention (not shown). Elderly doctors had the lowest initial scores, which improved during the program, but still remained the lowest. Doctors younger than 35 improved most during the voucher program. In contrast to the analyses mentioned above, individualdoctors arehere the unit of analysis. The results are reflected in first part of table 3.

Comparing the public, NGO and private clinics, the NGO clinics performed best before the intervention. During the intervention, the improvements observed were more pronounced among the public and private clinics, that is, the clinics with weakest initial performance. The performances became of a comparable quality during the intervention, but the improvements were not sustained after the intervention ceased (Second part of table 3).

## Discussion

Experiences of the SPs without vouchers illustrate the obstacles faced by adolescents in obtaining contraceptives through health services. Although all were eligible contraceptive users, a considerable number returned 'empty-handed'. For some important aspects the quality of care improved during the voucher program: more girls were involved in the method choice and more girls received a method. The improvements were more pronounced among providers and within clinics with the weakest initial performance. Shared decision-making and condom promotion were improvements that sustained after the program ended.

### Methodological considerations

No mention was made by any SP or doctor that a patient was "detected" as SP, meaning that the method was valid (face validity). Judged from the medical records of patients who went with a voucher to the clinic, the SPs performed their role correctly.

SP studies designed to measure differences usually limit themselves to record doctors' actions because inter-observer variability for criteria related to doctor-patient interaction is much higher [13,14,21,23]. Nevertheless, we incorporated the patients' perception in the criteria, because their subjective evaluation is probably the most important factor determining patient satisfaction [24], and decisive for future use of services. Our SPs were well instructed, relatively well educated, from a relatively homogeneous group, and were interviewed immediately after the consultation. As a result, we consider the differences found as indicative of real differences in performance.

We cannot exclude that the simulated patients were treated differently because of their somewhat higher level of education and higher age than the average voucher redeemer. However, accessing sexual and reproductive health care is difficult for many adolescents in Nicaragua, even for those who attended secondary school. Because the same group of girls participated in the pre- during and post assessment, the changes observed do not relate to their background.

Significant changes were foremost observed in criteria directly influenced by the program, such as how a decision on type of contraceptive method was taken and the improved delivery of methods. This may indicate that these criteria were the only ones influenced by the program. Because we compared clinics and not individual providers and the sample size was modest, it is also possible that less pronounced effects, or effects with lower inter-observer and/or inter-provider agreement, may not have been detected.

### The voucher program

The voucher program had a substantial impact on the number of women that left the clinic with a contraceptive method. This is in accordance with the programs instruction to give any women who requested a contraceptive method, a one month supply of that method with clear instructions on when to start (first day of the next menstruation) and to use condoms before. Patients were asked to return during the month following their menstruation and to relate their experiences. In this return visit, still covered by the voucher, patients could then receive a three months supply.

Clinics and doctors with weakest initial scores showed biggest improvements, suggesting they made serious efforts to comply with the program requirements. Unfortunately, most changes did not sustain beyond the program. Furthermore, even during the program there is room for further improvement. A longer intervention period and specific strategies to enhance quality improvements might realize more substantial and more sustainable changes. The voucher program carries this possibility. On one hand, the participating doctors were enthusiastic about the program, appreciated the experience, and were interested to improve their communication and other skills [25]. On the other hand, the approach can easily be strengthened by strategies such as interactive education in small groups [26] or supervision and self-assessment [27], which have proven to be effective in prompting sustained changes in daily practice.

Another strategy that could be combined with the voucher program is explicit use of the criteria for 'adolescent friendliness' [7]. Recently, a tool has been developed for assessing and improving reproductive health services for youth [12]. This assessment is best done by a small group of stakeholders, including adolescents, and can be used to grant clinics a label as 'adolescent friendly' or to develop an action plan for clinics to become adolescent friendly. Public display of such label signals to adolescents before entering the clinic that the staff will be receptive to their needs. A voucher program can limit inclusion to clinics and doctors that agree to conform to the standards required to be labelled 'adolescent friendly'. An additional and important advantage of such an approach would be that all adolescent patients are likely to benefit (and not only voucher bearers).

### The simulated patient method

Preparing and sending SPs, and recording their experience is the simplest part of the SP method. Their experiences provide valuable information about the actual performance of health services. The SP method, when openly employed, can furthermore be a useful support for regular feedback and identification of further training needs. It becomes more complicated when the experiences of SPs are to be converted into indicators to assess and evaluate changes in quality of care in a scientifically correct manner. This methodological challenge is insufficiently emphasized in the literature. Although the SP method is particularly recommended for evaluating programs with a focus on the interaction between patients and doctors, difficulties and solutions related to standardized assessment of the inherently subjective patient's opinion are not reported. This is an interesting field for future research in view of the importance of patients' perception in health care use.

Assessing quality improvements -including through the SP method- is complicated and requires highly trained human resources, time and financial resources. Although many health care interventions in developing countries aim to achieve quality improvements, programs are seldom supplied with sufficient funds to make such valuable and important evaluations possible.

## Conclusion

The adolescent voucher program seemed to have succeeded in increasing the accessibility of contraceptives through health services, and had the greatest impact on the quality of care in clinics with lowest initial quality levels. This is an important achievement, because if unwanted and untimely pregnancies are to be prevented, adolescents who engage in sexual relations need access to information and contraceptive methods.

Although in Nicaragua wide agreement exists that health care services should respond to these needs -as is explicitly outlined in norms and guidelines of the Ministry of Health [28] – implementation in daily practice lags well behind. The voucher program appears to be a relatively simple intervention able to improve important aspects of the quality of care delivered to adolescents. During the program adolescents received health care that better responded to their needs. Not all changes sustained after the program ceased. It is likely that implementation of the intervention over a longer period of time, with continued support to the providers, would be able to prompt a more sustained improvement in the quality of SRH care provided to adolescents.

The SP method proved to be a valuable and feasible method for program monitoring in a developing country. The results of this pilot study suggest that scaling up of the voucher program could be an effective strategy to make SRH care accessible, acceptable and appropriate for underserved teenagers.

## Abbreviations

AIDS Acquired Immune Deficiency Syndrome.

DFID The British Department for International Development

FP Family Planning

HIV Human Immune Deficiency Virus

ICAS instituto CentroAmerica de Salud, Central American health institute

NGO non-governmental organisations

RTI Reproductive tract infection

SP Simulated patient

SRH Sexual and reproductive health

STI Sexually transmitted infections

## Competing interests

The author(s) declare that they have no competing interests.

## Authors' contributions

LE M was involved in all stages of the process from conception and design of the survey, the analysis and interpretation of data. AC G was involved in the conception and design, the analysis and interpretation of the data, participated in writing and revising the paper critically for substantial intellectual content until the final approval. ADM K was involved in the statistical analysis and interpretation of the data, participated in writing and revising the paper critically for substantial intellectual. JA K gave support in the development of the research, the analysis and interpretation of the data, and contributed substantially in preparing the manuscript, revising it critically for substantial intellectual content. All authors read and approved the final manuscript.

## Pre-publication history

The pre-publication history for this paper can be accessed here:



## Figures and Tables

**Table 1:** Results related to contraception and STI/HIV prevention before, during and after the intervention

**Categories with their criteria**	**Before**	**During (with voucher)**		**After**	
	16 clinics (%)^1^	17 clinics (%)^1^	P^2 ^(14 pair)	15 clinics (%)^1^	P^3 ^(12 pair)
**A. Family planning, choice of method**					
1. Asks any question on the sexual relationship	9 (*56)*	10.7 (*63)*		4 (*27)*	
2. Presents minimal three contraceptive methods	12 (*75)*	10.6 (*62)*	0.09	10 (*67)*	
3. Does not create unnecessary barriers for the use of chosen methods	12 (*75)*	17.0(*100)*	0.08	10 (*67)*	
4. Decides on best method together with the girl	5 (*31)*	13.3 (*78)*	0.02	11 (*73)*	0.03
5. Patient is satisfied with explanations on FP methods	5 (*31)*	11.3 (*67)*		8 (*53)*	0.08
**Score 1**	**5.4**	**7.4**		**5.7**	
					
**B. Family planning, continuity in use**					
1. Patient left with a contraceptivemethod/receipt	8 (*50)*	16.0 (*94)*	0.01	12 (*80)*	0.10
2. Correct information is given on use of selected method	10 (*62)*	14.7 *(86)*		10.5 (*70)*	
3. New appointment was made	13 (*81)*	16.2 (*95)*		7 (*47)*	0.08
4. Health education material on FP is given	4.5 (*28)*	8.6 (*51)*		4.5 (*30)*	
5. Patient is satisfied with attention doctor	9.5 (*59)*	11.3 (*67)*		8.5 (*57)*	
**Score 2**	**5.6**	**7.9**		**5.7**	
					
**C. STI/HIV prevention**					
1. Discussed risks STI/HIV	10.5 (*66)*	13.9 (*82)*		13.5 (*90)*	
2. Gave correct information on prevention of STI/HIV	8 *(50)*	11.4 (*67)*		12 (*80)*	0.10
3. Gave health education material	5 (*31)*	11.0 (*65)*		3 (*20)*	
4. Patient satisfied with the explanations	5 (*31)*	8.1 (*48)*		6.5 (*43)*	
5. Promoted the use of condoms	8.5 (*53)*	11.9 (*70)*		14 (*93)*	0.03
6. Asked if patient had other concerns/asked for RTI/STI related complaints	6.5 (*41)*	5.1 (*30)*		5 (*33)*	
**Score 3**	**4.5**	**6.0**		**6.0**	

**Sum score 1 + 2 + 3**	**15.5 (sd 7.8)**	**20.9 (sd 6.9)**		**17.4 (sd 7.2)**	

FP Family Planning STI Sexually Transmitted Disease

^1^Column before: the information is based on 17 observations. Column during: the information is based on 30 observations. Column after: the information is based on 18 observations. When a clinic was visited more than once, the mean values of that clinic were calculated and considered

^2 ^the p value that is the result of the Wilcoxon Signed Ranks test (Paired design), comparing the scores of the clinics before and during the program, only p-values ≤ 0.10 are reflected.

^3 ^the p value that is the result of the Wilcoxon Signed Ranks test (Paired design), comparing the scores of the clinics before and after the program, only p-values ≤ 0.10 are reflected.

**Table 2:** Results related to the organisation of the clinic before during and after the intervention

**D. Organisation of the clinic**	**Before**	**During**	**P^2^**	**After**	**P^3^**
	16 clinics (%)^1^	17 clinics (%)^1^	(14 pair)	15 clinics (%)^1^	(12 pair)
1. Clean and appropriate	14 (*88)*	14.3 (*84)*		10.5 (*70)*	
2. Waiting time not more than 1 hour	14 (*88)*	13.9 (*82)*		13.5 (*90)*	
3. Privacy maintained during consultation	11 (*69)*	12.5 (*74)*		15 (*100)*	0.08
4. No interruptions during consultation	12 *(13)*	13.3 (*78)*		13 (*87)*	
5. Consultation time of at least a quarter of an hour	12 (*15)*	14.8 (*87)*		10.5 (*70)*	
**Score 4**	**7.9**	**8.1**		**8.3**	

^1 ^Column before: the information is based on 17 observations. Column during: the information is based on 30 observations. Column after: the information is based on 18 observations. When a clinic was visited more than once, the mean values of that clinic were calculated and considered

^2 ^the p value that is the result of the Wilcoxon Signed Ranks test (Paired design), comparing the scores of the clinics before and during the program, only p-values ≤ 0.10 are reflected.

^3 ^the p value that is the result of the Wilcoxon Signed Ranks test (Paired design), comparing the scores of the clinics before and after the program, only p-values ≤ 0.10 are reflected.

**Table 3:** Influence of doctors and clinics characteristics on their performances before during and after the intervention.

**Scores ^1^**	**Before**			**During (with voucher)**			**After**		
**Gender ^2^**	**N = 17**	**Mean**	**p^3^**	**N = 24**	**Mean**	**p^3^**	**N = 17**	**Mean**	**p^3^**
Women	9	19.7 (sd 8.0)		16	22.8 (sd 6.5)		11	20.5 (sd 5.6)	
Male	8	9.7 (sd 2.9)	0.01	8	16.4 (sd 7.1)	0.05	6	12.6 (sd 5.6)	0.03
**Age group ^2^**			**p^4^**			**P^4^**			**p^4^**
30–34	6	14.8 (sd 8.9)		7	24.2 (sd 5.3)		6	18.7 (sd 6.1)	
35–39	7	17.6 (sd 8.7)		12	19.8 (sd 7.3)		7	21.2 (sd 4.6)	
40 +	4	10.8 (sd 3.1)		5	17.7 (sd 8.6)		4	10.1 (sd 4.7)	0.04

**Type of clinic ^5^**	**N = 16**		**P^4^**	**N = 17**		**P^4^**	**N = 15**		**P^4^**
Public	4	12.5 (sd 8.0)		3	22.2 (sd 10.3)		2	14.8 (sd 2.9)	
NGO	8	18.4 (sd 8.6)		10	20.6 (sd 4.9)		9	20.7 (sd 6.2)	
Private	4	12.9 (sd 5.1)		4	22.1 (sd 6.3)		4	11.3 (sd 7.2)	

^1 ^Mean sum score of categories I to III reflecting the consultation

^2 ^During the voucher programme some doctors were visited more than once by SPs, but for this analysis only the first visits were considered. After the intervention 1 SP was seen by a Nurse.

^3 ^Outcome of the Mann Whitney test (comparing the mean sum score of men and women) only p-values ≤ 0.10 are reflected.

^4 ^Outcomes of the Kruskall Wallis test, only p-values ≤ 0.10 are reflected.

^5 ^For each clinic the mean score is considered. Column 'before ' is based on 17 observations, column 'during' on 30 observations and column 'after' on 18 observations, only p-values ≤ 0.10 are reflected.
